# Lymphoma and the Kidney: A Kidney Biopsy Teaching Case

**DOI:** 10.1016/j.xkme.2020.06.011

**Published:** 2020-08-08

**Authors:** Vincenzo L’Imperio, Mattia Rossi, Afu Abdul, Satyen R. Mehta, Aaron C. Shaver, Agnes B. Fogo

**Affiliations:** 1 Department of Medicine and Surgery, Pathology, San Gerardo Hospital, University of Milano-Bicocca, Monza, Italy; 2 Department of Pathology, Microbiology and Immunology, Vanderbilt University Medical Center, Nashville, TN; 3 Renal Unit, Department of Medicine, University and Hospital of Verona, Verona, Italy; 4 Nephrology, Harbin Clinic, Cartersville, GA; 5 Hematology, Northwest Georgia Oncology Center, Cartersville, GA; 6 Division of Hematopathology, Department of Pathology, Microbiology, and Immunology, Vanderbilt University Medical Center, Nashville, TN

**Keywords:** Kidney pathology, hematopathology, kidney limited lymphoma, DLBCL, kidney biopsy

## Abstract

Lymphomatous infiltration of kidney parenchyma is a frequent complication of systemic hematologic malignancies and often shows subtle clinical presentation. Diffuse large B-cell lymphoma represents the most frequent form involving the kidney, with advanced stage at diagnosis, poor outcome, and risk for central nervous system relapse if not adequately treated. Kidney biopsy can provide specific and early detection of these cases, helping in the differential diagnosis with more frequent entities. Finally, further hematologic workup (bone marrow biopsy, complete blood cell count, and positron emission tomography) can distinguish secondary involvement of the kidney from the rarer kidney-limited forms, especially in patients without a previous diagnosis of lymphoma. Making a prompt and correct diagnosis directs the management of these cases and may improve the outcome, as described in the present report.

## Introduction

Lymphomatous infiltration of the kidney parenchyma is a frequent complication of hematologic disorders, present in ∼34% of patients with lymphoma in a large series.^1^ However, only 14% of cases are detected before death,^1^ and this underdiagnosis could be due to the subtle clinical presentation of kidney involvement, with only 0.5% of such cases showing acute kidney injury.^2^ Few data are available regarding the incidence of kidney infiltration for the different lymphoma subtypes. Diffuse large B-cell lymphoma (DLBCL) is the most frequent involving the kidney, showing advanced stage at diagnosis, poor outcome, and risk for central nervous system relapse if not promptly detected and adequately treated.^3^

Although the detection of parenchymal swelling/enlargement on imaging can be of help, kidney biopsy is mandatory in these cases, particularly in patients with no prior lymphoma diagnosis.^4^ Finally, hematologic workup with appropriate staging (bone marrow biopsy, complete blood cell count, and positron emission tomography [PET]) can allow the distinction of secondary kidney involvement in systemic disease from the rarer primary kidney lymphoma, especially in patients without a history of hematologic disease.^5^ This can direct the management of these cases and may improve their outcome. We report a case of systemic DLBCL presenting with apparent initial kidney-limited involvement in which diagnosis of the kidney component led to thorough clinical and radiographic staging that identified the full extent of disease, with discussion of the possible interpretative pitfalls in the histologic assessment.

## Case Report

A white man in his 50s with a history of hypertension, coronary artery disease, and obesity was referred to nephrology for evaluation of acute kidney injury in the setting of nausea, vomiting, and abdominal pain.

### Initial Laboratory Data

Laboratory data showed the following values: serum creatinine, 5.5 mg/dL; estimated glomerular filtration rate, 11 mL/min/1.73 m^2^; urinary protein-creatinine ratio, 0.98 mg/mg; no hematuria; and blood pressure, 164/112 mm Hg. Kidney ultrasonography and computed tomography (CT) of the chest, abdomen, and pelvis showed mild enlargement of the kidneys (13.3 cm on the right and 12.5 cm on the left) without other significant findings. Because the clinical workup failed to determine a possible cause of the sudden loss of kidney function, percutaneous kidney biopsy was performed.

### Kidney Biopsy

**Figure 1:**
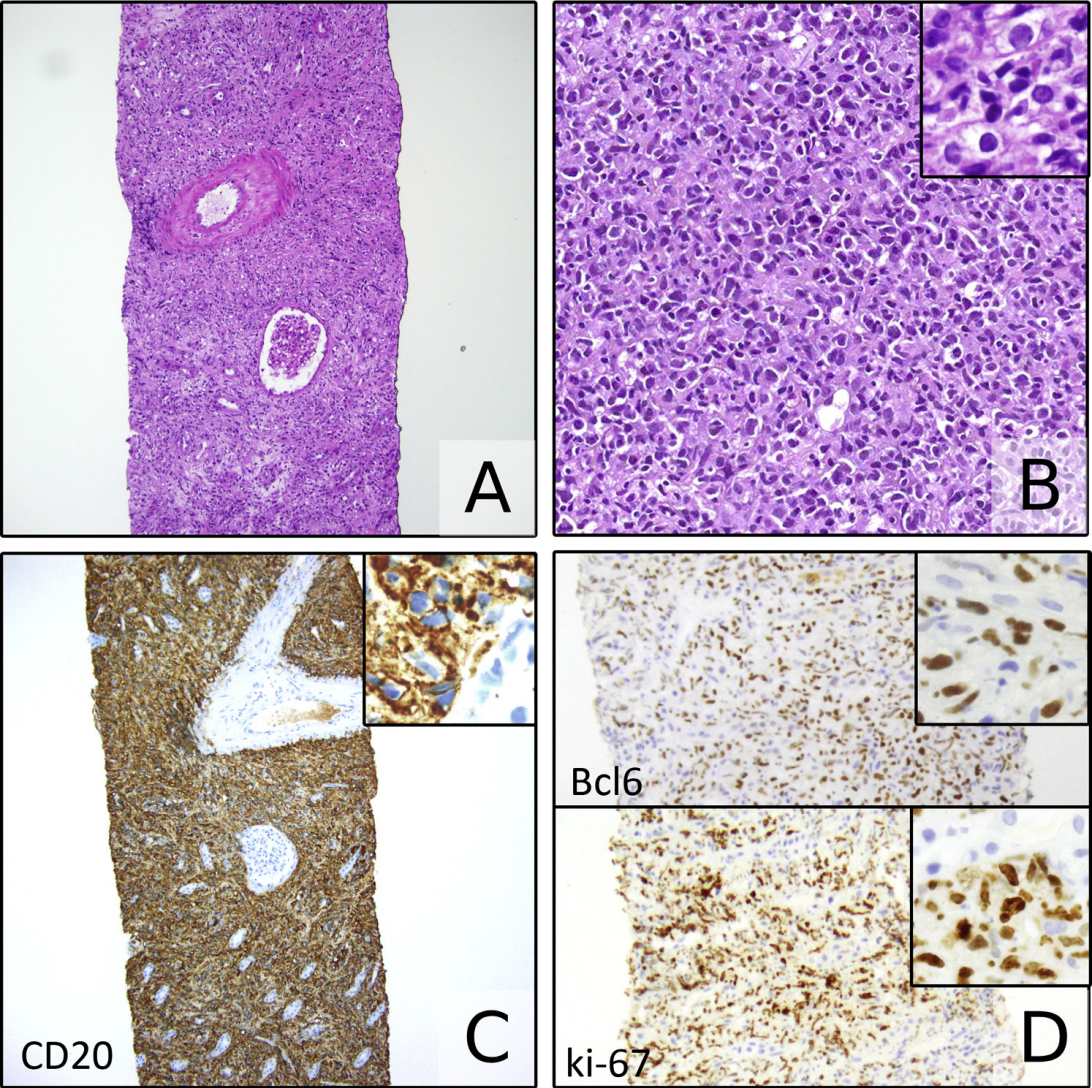
An extensive infiltrate obliterated the kidney parenchyma (A, hematoxylin and eosin; original magnification, ×50), composed of a mixture of small lymphoid-appearing cells, some spindly cells, and cells with enlarged atypical nuclei (B, hematoxylin and eosin; original magnification, ×200; inset; original magnification, × ∖400). Atypical cells were diffusely positive for CD20 (C) and Bcl6 (D, top of the picture) with 70% to 80% Ki-67 positivity (D, bottom of the picture; original magnification, ×10; insets; original magnification, × 40).

Kidney biopsy showed 4 glomeruli that were unremarkable. Immunofluorescence and electron microscopy showed no deposits. Kidney parenchyma was obliterated by an extensive mixed lymphoid infiltrate composed of small cells, spindled cells, and a predominance of large lymphoid cells with abnormal nuclei (Fig 1A). The infiltrating cells were diffusely positive for B-cell markers CD20 (Fig 1B), PAX5 (Paired Box 5), and CD79a, characteristic of a large B-cell lymphoma. There was also positivity for Bcl6 (Fig 1C) and negativity for CD10 and MUM1 (Multiple Myeloma 1), indicating germinal center phenotype by the Hans classification.^6^ The Ki-67 proliferative rate was 70% to 80% (Fig 1D). The abnormal cells were negative for Epstein-Barr encoding region (EBER) in situ hybridization, BCL2, c-Myc (<40%), CD138, CD30, CD3, and CD68 (markers of possible viral status, differentiation, and grade, as discussed later). Because c-Myc and BCL2 were negative, fluorescence in situ hybridization for the detection of rearrangements of these loci has not been performed. There were scattered background CD3- and CD68-positive cells.

### Diagnosis

Taken together, these findings were consistent with DLBCL, not otherwise specified, although a large-cell transformation of an underlying systemic B-cell neoplasm could not be fully excluded.

After the preliminary kidney biopsy results, repeat CT of the chest, abdomen, and pelvis demonstrated unchanged bilateral kidney prominence without additional findings. CT of the neck showed mildly enlarged tissue adherent to the inferior aspect of both parotid glands, measuring 1.1×1.7 cm on the right and 1.2×1.8 cm on the left, attributable to abnormal lymph nodes. The patient underwent subsequent hematologic workup with unremarkable findings for complete blood cell count (white blood cell count, 8.2 ×10^3^/μL; red blood cell count, 3.93 ×10^6^/μL; hemoglobin, 11 g/dL; hematocrit, 32.3%; platelet count, 211 ×10^3^/μL; neutrophil count, 6.13 ×10^3^/μL; lymphocyte count, 1 ×10^3^/μL; monocyte count, 1 ×10^3^/μL; eosinophil count, 0.02 ×10^3^/μL; and basophil count, 0.01 ×10^3^/μL). A trephine bone marrow biopsy demonstrated no evidence of bone marrow involvement by lymphoma, further suggesting a possible form of kidney-limited lymphoma. However, PET performed after the kidney biopsy report demonstrated bilateral intraparotid positivity (proved to be benign Warthin tumor by biopsy) and systemic PET positivity in different sites (kidneys and adrenal glands bilaterally, thyroid gland, spleen, and throughout the entire skeleton), ascribed to systemic involvement by the patient’s lymphoma.

### Clinical Follow-up

The patient was treated with rituximab and CHOP (cyclophosphamide, doxorubicin, vincristine, and prednisone) for his lymphoma. The patient required dialysis for 2 months. After 6 cycles of chemotherapy, PET showed a complete response with regression in all sites and the patient experienced an improvement in kidney function, with serum creatinine level of 2.2 mg/dL and estimated glomerular filtration rate of 32 mL/min/1.73 m^2^.

## Discussion

The diagnosis of kidney lymphoma can be challenging due to the subtle clinical presentation, ranging from silent kidney masses that can mimic renal cell carcinoma, kidney abscesses, or other kidney tumor metastasis by imaging studies, less frequently presenting with acute kidney injury or subnephrotic proteinuria, often lacking weight loss or flank pain.^7^ This can lead to diagnostic delay and increased risk for poor outcome of these cases. In some series, DLBCL represents the most frequent form of lymphoma involving the kidney, with bilateral forms accounting for 44% of cases.^3^ Kidney biopsy typically shows extensive or complete obliteration of the parenchyma (Box 1), a shared feature of both reactive and neoplastic processes affecting the kidney.

Box 1 **Key Pathology Findings in Kidney Lymphoma**

**Table:** 

1. Extensive or complete obliteration of the parenchyma
2. Dense infiltrate mainly composed of a mixture of small lymphoid-appearing cells, some spindly cells, and cells with enlarged atypical nuclei
3. Diffuse positivity of the neoplastic elements for B-cell lineage antigens in immunohistochemistry (eg, CD20, CD79a, and PAX5)
4. High proliferative rate assessed through Ki-67 immunohistochemistry (usually >40% in DLBCL cases)
5. Variable expression of additional immunohistochemical markers to define the cell of origin (CD10, Bcl6, MUM1) or identify high-grade lymphomas (c-Myc and Bcl2)

Abbreviations: DLBCL, diffuse large B-cell lymphoma; MUM1, multiple Myeloma 1; PAX5, Paired Box 5.

Several conditions can cause infiltrates of lymphoid cells in the kidney but do not cause obliteration of kidney architecture. Acute interstitial nephritis is characterized by a polymorphic leukocytic interstitial infiltrate composed of B and T lymphocytes, plasma cells, macrophages, and even neutrophils associated with edema and tubulitis. Acute pyelonephritis can cause kidney swelling and shows patchy inflammation with lymphocytes, plasma cells, and neutrophils with the presence of “neutrophilic plugs” in the tubular lumina. Malakoplakia and xanthogranulomatous pyelonephritis (XGP) are chronic inflammatory conditions caused by repeated infections and characterized by diffuse and destructive histiocytic infiltrates, with CD68 positivity of these infiltrating cells. In malakoplakia, there are typical Michaelis-Gutmann bodies (concentric basophilic inclusions representing mineralized phagosomes composed of iron and calcium deposits), whereas XGP shows numerous foam cells. Finally, systemic inflammatory conditions, such as immunoglobulin G4 (IgG4)-related disease, can rarely involve the kidney and present with unilateral or bilateral masses. Biopsy shows a dense “storiform” fibrosis, increased IgG4-positive plasma cell infiltrate, and frequently associated granular tubular basement membrane deposits.

Among the neoplastic processes, systemic hematologic disorders can lead to diffuse infiltration of kidney parenchyma. The most frequent forms are represented by lymphomas (both low-grade B-cell non-Hodgkin lymphoma and DLBCL), followed by acute lymphoblastic leukemia and, rarely, myeloid neoplasms (such as acute myeloid leukemia and Langerhans and non-Langerhans histiocytosis).^8^^,^^9^ All these are characterized by the presence of monotypic neoplastic elements and the use of adequate immunohistochemical stains can aid in the differential diagnosis. Epstein-Barr virus–positive DLBCL is characterized by in situ hybridization positivity for EBER in neoplastic cells,^10^ whereas plasmablastic lymphoma shows typical CD138 positivity.^11^ In anaplastic lymphoma (both ALK-positive and -negative forms), neoplastic elements are CD30-positive.^12^ For prognostic purposes, it is important to note that immunohistochemical markers (eg, CD10, Bcl6, and MUM1) can indicate the type of cell of origin in DLBCL and some (eg, Bcl2 and c-Myc) can have a role in differentiating not otherwise specified forms from high-grade lymphomas.^13^

The evidence of lymphoma on kidney biopsy leads to an important differential diagnosis between the rare kidney-limited form and systemic lymphoproliferative disorder involving the kidney. The absence of extrarenal localization with appropriate clinical staging could be indicative of primary kidney lymphoma, which accounts for ∼0.7% of all extranodal lymphomas in North America,^14^ the most frequent subtype being DLBCL.^15^ These forms show a poor outcome with a reported 1-year mortality rate as high as 75%^16^ and a 5-year survival rate of only 40% to 50%, with median survival time of 8 months to 3 years.^17^ However, the detection of extrarenal sites involved by lymphoma may be revealed by comprehensive staging and thus lead to a diagnosis of systemic lymphoproliferative disorder presenting with kidney involvement, with important consequences for the prognosis and therapy, as demonstrated in this case. Because of the important prognostic ramifications, this case report illustrates the vital importance of careful evaluation of the kidney biopsy and appropriate recognition of the differential diagnosis to guide the subsequent hematologic evaluation.
